# Increasing the Endoplasmic Reticulum Pool of the F508del Allele of the Cystic Fibrosis Transmembrane Conductance Regulator Leads to Greater Folding Correction by Small Molecule Therapeutics

**DOI:** 10.1371/journal.pone.0163615

**Published:** 2016-10-12

**Authors:** W. Joon Chung, Jennifer L. Goeckeler-Fried, Viktoria Havasi, Annette Chiang, Steven M. Rowe, Zackery E. Plyler, Jeong S. Hong, Marina Mazur, Gary A. Piazza, Adam B. Keeton, E. Lucile White, Lynn Rasmussen, Allan M. Weissman, R. Aldrin Denny, Jeffrey L. Brodsky, Eric J. Sorscher

**Affiliations:** 1 Gregory Fleming James Cystic Fibrosis Research Center, University of Alabama at Birmingham, Birmingham, Alabama, United States of America; 2 Department of Neurobiology, University of Alabama at Birmingham, Birmingham, Alabama, United States of America; 3 Department of Biological Sciences, University of Pittsburgh, Pittsburgh, Pennsylvania, United States of America; 4 Department of Medicine, University of Alabama at Birmingham, Birmingham, Alabama, United States of America; 5 Department of Cellular, Developmental and Integrative Biology, University of Alabama at Birmingham, Birmingham, Alabama, United States of America; 6 Oncologic Sciences, USA Mitchell Cancer Institute, University of South Alabama, Mobile, Alabama, United States of America; 7 Drug Discovery Division, Southern Research Institute, Birmingham, Alabama, United States of America; 8 Center for Cancer Research, National Institutes of Health, Frederick, Maryland, United States of America; 9 Worldwide Medicinal Chemistry, Pfizer, Cambridge, Massachusetts, United States of America; 10 Department of Pediatrics, Emory University, Atlanta, Georgia, United States of America; Ohio State University, UNITED STATES

## Abstract

Small molecules that correct the folding defects and enhance surface localization of the F508del mutation in the Cystic Fibrosis Transmembrane conductance Regulator (CFTR) comprise an important therapeutic strategy for cystic fibrosis lung disease. However, compounds that rescue the F508del mutant protein to wild type (WT) levels have not been identified. In this report, we consider obstacles to obtaining robust and therapeutically relevant levels of F508del CFTR. For example, markedly diminished steady state amounts of F508del CFTR compared to WT CFTR are present in recombinant bronchial epithelial cell lines, even when much higher levels of mutant transcript are present. In human primary airway cells, the paucity of Band B F508del is even more pronounced, although F508del and WT mRNA concentrations are comparable. Therefore, to augment levels of “repairable” F508del CFTR and identify small molecules that then correct this pool, we developed compound library screening protocols based on automated protein detection. First, cell-based imaging measurements were used to semi-quantitatively estimate distribution of F508del CFTR by high content analysis of two-dimensional images. We evaluated ~2,000 known bioactive compounds from the NIH Roadmap Molecular Libraries Small Molecule Repository in a pilot screen and identified agents that increase the F508del protein pool. Second, we analyzed ~10,000 compounds representing diverse chemical scaffolds for effects on total CFTR expression using a multi-plate fluorescence protocol and describe compounds that promote F508del maturation. Together, our findings demonstrate proof of principle that agents identified in this fashion can augment the level of endoplasmic reticulum (ER) resident “Band B” F508del CFTR suitable for pharmacologic correction. As further evidence in support of this strategy, PYR-41—a compound that inhibits the E1 ubiquitin activating enzyme—was shown to synergistically enhance F508del rescue by C18, a small molecule corrector. Our combined results indicate that increasing the levels of ER-localized CFTR available for repair provides a novel route to correct F508del CFTR.

## Introduction

Cystic fibrosis is a genetic disease caused by mutations in the gene encoding CFTR [1]. The predominant cause of morbidity and mortality in CF is attributable to chronic lung disease, although clinical manifestations of CF can also include pancreatic damage, hepatic injury, infertility and other exocrine dysfunction. The most prevalent CFTR mutation involves deletion of phenylalanine at CFTR position 508 (F508del), which leads to misfolding and subsequent premature degradation by the Endoplasmic Reticulum Associated Degradation (ERAD) pathway [2–7]. Because ~90% of CF patients carry at least one F508del allele, the identification of small molecules that correct folding defects and enhance surface localization of F508del CFTR has been actively pursued. In the past, correctors of F508del (e.g., C18 and Vertex-809, or lumacaftor) were identified by high throughput functional assays that monitor CFTR-dependent anion transport [8]. Unfortunately, restoration of F508del CFTR function has been difficult to achieve as monotherapy, although hundreds of thousands of discrete molecules have been analyzed for this purpose by unbiased high throughput screening [9, 10]. Indeed, a combination of lumacaftor, which increases the activity of F508del CFTR to ~25% of WT levels in cell culture [8], only increases lung function by ~4% in F508del homozygotes when combined with a potentiator that helps to open the channel [11].

Improved F508del rescue may also have particular relevance to the large number of patients carrying a compound heterozygous genotype (i.e., those with one F508del allele and a different mutation on the second allele), for whom current pharmaco-corrective treatments are insufficient. Potential barriers to F508del correction from this perspective include the need to overcome multiple checkpoints in the CFTR folding pathway, each of which may abrogate biogenesis. For example, poor structural or temporal access of small molecules to CFTR folding intermediates, robust ERAD, enhanced endocytosis of F508del CFTR when corrected and resident at the plasma membrane, and/or the complex proteostatic network that governs CFTR maturation may each be difficult to overcome with a single agent [12–14]. These and perhaps other fundamentally distinct abnormalities caused by F508del further emphasize challenges to small molecule repair.

Defects in F508del CFTR mRNA integrity have also been noted. Specifically, the utilization of F508del mRNA may be severely diminished compared to WT [15, 16]. Even when levels of the F508del and WT mRNA are similar, the F508del mRNA also appears to be misfolded, which diminishes F508del CFTR protein synthesis. Identifying molecules capable of overcoming defects involving both CFTR protein conformation and mRNA structure, for example by using high throughput compound library screening, may therefore be quite challenging.

We and others have hypothesized that insufficient levels of F508del Band B—the immature, ER resident, form of the channel—significantly contribute to poor efficiency of pharmacologic rescue. As noted above, although a clinically approved agent (lumacaftor) and a structural analog, C18, can augment maturation of F508del CFTR to ~25% of WT [8, 17], this magnitude of rescue is insufficient to restore pulmonary function among patients harboring a single F508del allele. In this report, therefore, we designed a series of experiments to address the following questions. First, can a compound library screen be used to identify small molecules that improve F508del CFTR correction to Band C, the post-ER glycoform, in human epithelial cells? Second, will small molecules that enhance the “foldable” CFTR pool (i.e., Band B) exhibit synergy with analogues of lumacaftor? Third, can F508del CFTR degradation be interrupted or stalled in a fashion that increases the amount of mutant protein available for repair by a CFTR corrector? Our findings demonstrate that increasing the foldable, ER localized pool of F508del Band B allows for synergistic correction by compounds such as C18 or lumacaftor. Based on estimates that as little as a three-fold increase in lumacaftor correction may offer substantial clinical benefit to CF patients [18], this approach offers a means by which F508del folding defects might be overcome in a clinically meaningful fashion.

## Materials and Methods

The studies conducted for this paper were deemed "not human subject research" by the University of Alabama at Birmingham (UAB) Institutional Review Board. All cells and cell models were obtained under regulatory supervision and fully de-identified.

### Cell culture conditions and CFTR expression analysis

Cells were maintained in a 37°C humidified incubator with 5% CO_2_. HeLa, HEK 293, and CFBE41o− cell lines were obtained from the American Type Culture Collection (ATCC, Manassas, VA) or as described previously [19]. HeLa and HEK 293 cells were grown in DMEM (Dulbecco’s modified Eagle’s medium, Invitrogen, Grand Island, NY) supplemented with 10% (v/v) FBS (fetal bovine serum, Invitrogen). CFBE41o− cells were maintained in MEM (minimal essential medium; Invitrogen) supplemented with 10% (v/v) FBS. For experiments requiring polarized cells, CFBE41o—F508del and CFBE41o—WT cells were seeded on 6 mm diameter Transwell filters (Costar, Corning, Tewksbury, MA). Primary airway epithelial cells were initially expanded in BEBM (Lonza, Basel, Switzerland) supplemented with BEGM single shots (Lonza), and cultured in differentiating media as described in [20]. Primary airway epithelial cells were obtained by the UAB Cystic Fibrosis Tissue Procurement Core after obtaining written informed consent, and with approval of the UAB Institutional Review Board.

Total CFTR was analyzed by SDS/PAGE (6% gel) and western blotting. CFTR was detected using a 1:1 mixture of mouse monoclonal antibodies 570 and 596 (Cystic Fibrosis Foundation Therapeutics) and Alexa488- conjugated anti-mouse IgG (Invitrogen). Chemiluminescence was performed with SuperSignal West Femto Maximum Sensitivity substrate (Thermo Scientific, Waltham, MA) and analyzed using Chemidoc XRS (Bio-Rad, Hercules, CA).

CFTR mRNA levels were analyzed with qRT-PCR as described previously [21]. Total RNA was isolated using the RNeasy mini kit (Qiagen, Valencia, CA). Quantitative real-time PCR was performed using the ABI StepOnePlus sequence detection system (Applied Biosystems, Foster City, CA).

### Transepithelial Conductance measurements

FRT cells (obtained from Dr. M. Welsh, University of Iowa, Iowa City, IA) stably transduced with F508del CFTR were grown on permeable supports (3378; Costar) in Coon’s modified media containing 11.5g/l of F12 Ham nutrient mixture (Sigma-Aldrich, St. Louis, MO), 2.68g/l sodium bicarbonate, and 5% FBS. Transepithelial conductance of the cells was measured using a 24 channel current clamp (EP-Devices, Bertum, Belgium) equipped robot (PrecisePlace 2,300 Robot; Precise Automation Inc, La Jolla, CA) [22, 23]. Cells were bathed in FBS and sodium bicarbonate-free media and treated first with 10 μM amiloride followed by an agonist (20 μM forskolin), and then with a CF inhibitor 172 (10 μM; Sigma-Aldrich) to block CFTR dependent conductance.

### Ussing chamber measurements

CFBE41o-cells expressing WT or F508del CFTR were seeded onto permeable supports (Costar) after coating with fibronectin [24]. Cells were grown to confluence and transferred to an air—liquid interface, after which they were mounted in modified Ussing chambers. Monolayers were initially bathed on both sides with identical Ringers solution containing (in mM): 115 NaCl, 25 NaHCO_3_, 2.4 KH_2_PO_4_, 1.24 K_2_HPO_4_, 1.2 CaCl_2_, 1.2 MgCl_2_, 10 D-glucose (pH 7.4) and vigorously stirred and gassed with 95%O_2_: 5% CO_2_ at 37°C. Short-circuit current (Isc) was obtained using an epithelial voltage clamp. The mucosal bathing solution was changed to a low Cl^-^ solution containing (in mM): 1.2 NaCl, 115 Na gluconate, plus 100 μM amiloride followed by addition of agonists (20 μM forskolin, 50 μM genistein, and/or 10 μM ivacaftor) to the mucosal surface. CF inhibitor 172 (10 μM; Sigma-Aldrich, St. Louis, MO) was added to the bathing solution at the conclusion of each experiment to block CFTR-dependent Isc.

### Compound library screening

To perform high content microscopy screening, HeLa cells with stable expression of F508delCFTR [19] were cultured in 96-well PE ViewPlates (Perkin Elmer, Waltham, MA) for one day prior to well-by-well addition of two thousand known bioactive compounds from the NIH Roadmap Molecular Libraries Small Molecule Repository. Ten mM DMSO stocks of compounds were diluted 500-fold in growth medium, then added in equal volume to assay plates to yield a final screening concentration of 10 μM.

After a 24h treatment period, cells were fixed, permeabilized, and labeled with 3G11 antibody, which recognizes intracellular CFTR epitopes in NBD1, then visualized with AlexaFluor488 anti-mouse conjugate and DRAQ5 fluorescent DNA label (Axxora, Farmingdale, NY). Nine microscopic fields per sample well were then imaged using the Evotec Opera^™^ QEHS automated confocal microscope equipped with a 20x magnification Olympus objective lens (Perkin Elmer). Automated analysis of the resulting images was performed using Acapella^™^ (Perkin Elmer) software as follows. First, high contrast labeling of cellular nuclei by DRAQ5 enabled rapid image segmentation by intensity threshold. Corresponding cytoplasm and cell margins associated with each nucleus were subsequently defined by image threshold of lower contrast cytoplasmic / RNA staining of DRAQ5. Further segmentation of images into regions of interest for analysis included plasma membrane (a 2 pixel wide region corresponding to cell margin) and perinuclear region (a 5 pixel wide region encircling the outside of the nuclear mask). Three criteria were used to track expression and subcellular localization of: 1) F508del CFTR signal in the region of interest, designated as the plasma membrane (PM); 2) F508del CFTR signal in the perinuclear region of the cell, as a proxy for the ER; and 3) fluorescence signal across the entire cell (total) to monitor overall levels of F508del CFTR protein. Each measurement was expressed as the mean fluorescent intensity for all cells imaged in 9 microscopic fields per sample.

A high throughput 384-well microplate based fluorescence assay was also developed using HeLa cells stably expressing F508del CFTR. Cells were maintained in cell culture media (High Glucose DMEM (Gibco, Themo Fisher Scientific Inc., Waltham, MA) supplemented w/ 10% FBS (Gibco)). For the assay, cells were trypsinized (0.25% Trypsin-EDTA solution (Gibco)) and resuspended in assay media (cell culture media + 1% Pen/Strep (Gibco)) at a density of 320,000 cells per ml. Cells were dispensed to the assay plates (Corning 3712) in 25 μl using a Matrix Wellmate (Thermo Scientific). Plates were incubated overnight at 37°C, 5% CO_2_ and high humidity. The next day, compounds were prepared by making a dilution in assay media to a concentration of either 150 μM or 60 μg/ml (6x) depending on the library being screened. Next, 5 μl of the diluted compound was transferred to the assay plate containing the cells. Final conditions were: test compounds 25 μM or 10μg/ml, control drug ALLN (Calbiochem, San Diego, CA) 50 μM, control drug Hyamine (Sigma-Aldrich) 100 μM or DMSO 0.5% in all wells including cell controls. Assay plates were incubated for 24 hrs 37°C, 5% CO_2_ and high humidity. Following incubation with the compounds, the relative cell number was determined by adding 3μl of alamarBlue (TREK Diagnostics, Thermo Scientific) and incubating the plates at 37°C, 5% CO_2_ and high humidity, until cell control values reached ~4 million, as measured with a fluorescent intensity protocol on an Envision multimode plate reader (Perkin Elmer) (excitation 535 nm, emission 595 nm wavelengths). Following the alamarBlue read, media was removed and the cells were fixed with the addition of 13μl of 4% paraformaldehyde in PBS (pH 7.4) and incubated for 10 min at room temperature. Plates were washed twice with PBS to remove the fixative. Cells were permeabilized with the addition of blocking solution (PBS, 0. 1% Triton X-100, 10mg/ml BSA) and incubated at room temperature for 30 min. The blocking solution was removed and the primary antibody mixture was added in 13ul (Antibodies UNC570 and UNC596 (CFF Therapeutics, Inc., Bedford, MA)) at a 1/5000 dilution of each antibody in blocking solution. Plates were then incubated overnight at 4°C. Primary antibody was removed by washing three times with 50 μl of wash solution (PBS, 0.1% Triton X-100) with at least 5 min between wash cycles. Secondary antibody (Alexa 488 anti-mouse IgG Invitrogen) was then added in a volume of 13μl (1/1000 dilution in blocking solution) and incubated for two hrs at room temperature in the dark. To remove excess secondary antibody, plates were washed three times with 50 μl of wash solution and one time with 50 μl of PBS. Plates were read from below using a fluorescent intensity protocol on an Envision multimode plate reader (485 nm excitation and 535 nm emission wavelengths). The proteasome inhibitor ALLN was used as the positive control and Hyamine was used as a viability control on all microtiter plates. In-plate controls were used to normalize the data and results were reported as fold increase above the cell control and normalized to relative cell number derived from the alamarBlue data. Data were analyzed as follows: 1) Immunostain assay: fold increase = DataValue/Median cell control; 2) Cell Viability Assay: % viability = 100* (DataValue-median Hyamine control)/(Median cell control-median Hyamine control), Viability = (DataValue-median Hyamine control)/(Median cell control-median Hyamine control); 3) Normalized Fold Increase: Fold Increase/Viability.

### Statistical Analysis

For Isc, descriptive statistics (mean, SD, and SEM) and unpaired t tests (combined ΔIsc by forskolin and agonist) were performed using Microsoft Excel (Seattle, WA). All statistical analysis was two sided and performed at a 5% significance level (i.e., α = 0.05). Error bars represent SEM.

### Computational Methods

Protein and ligand preparation: The X-ray structure of the E1 ubiquitin activating enzyme (PDB code 3CMM) was used to model binding by PYR-41. Since this is an apo crystal structure, water molecules around the binding pocket near Cys 600 were deleted. The hydrogens to all heavy atoms were added with protein preparation wizard in the Maestro suite version 9.8 (Schrödinger, Portland, Oregon; http://www.schrodinger.com/) running under the Linux RedHat Enterprise WS OS. Atom and bond types were assigned, and the positions of hydrogens were optimized with OPLS2.1 force field. PYR-41 was first drawn in 2D in chemdraw and transferred into the Maestro suite. The addition of hydrogen and the 3D coordinates were developed in Maestro. The appropriate protonation check was performed using Epik software version 2.7 in the ligand preparation wizard at a physiologically relevant pH of 7.4.

Docking and druggability: PYR-41 was docked into the E1 ubiquitin activating enzyme using Glide version 6.2 (Schrödinger, LLC) running in a Linux environment. Ligand poses were analyzed using Maestro suite. For druggability analysis, code was developed to perform the maximal binding affinity calculation [25]. SiteMap druggability evaluation was performed in version 3.0 (Schrödinger, LLC; [26]).

## Results

### A diminished pool of F508del CFTR may hamper drug discovery efforts

**Fig 1:**
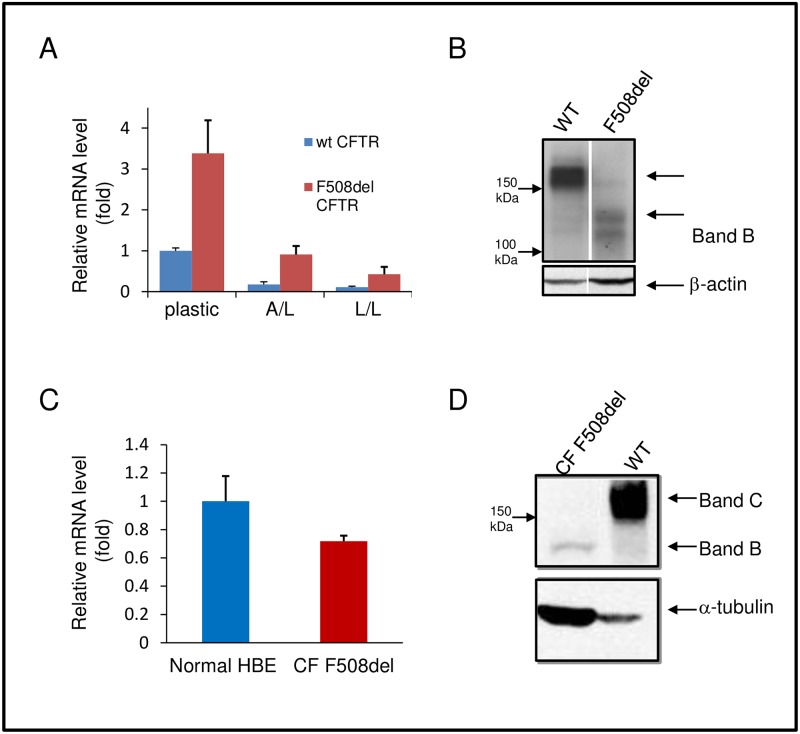
CFTR expression in airway epithelial cells. (A, B) Levels of mRNA (A), and protein (B) in human bronchial epithelial cells expressing wild type (WT) or F508del CFTR. Cells were grown on plastic, as polarizing cell monolayers on permeable supports using an air/liquid (A/L) interface, or on the same supports with overlying liquid (liquid/liquid, L/L). Mature, fully glycosylated CFTR (Band C), and the ER localized glycoform (Band B) are depicted for air/liquid interface culture, which is most representative of physiologic conditions. (C, D) Steady state levels of CFTR mRNA (C) and protein (D) in primary airway epithelial cells grown at an air/liquid interface and isolated from non-CF or F508del homozygous individuals. mRNA levels were normalized against CFBE cells expressing WT CFTR (A) or non-CF human primary airway cells (C) in fold difference. N ≥ 3 samples per condition. Representative western blots in (B) and (D) are shown.

Few cell lines express sufficient levels of endogenous F508del CFTR to permit detailed biochemical analysis of the mutant protein or to allow high throughput compound library screening (HTS) for drug discovery. In contrast, recombinant cell lines are available that express detectable F508del CFTR [24, 27]. For example, CFBE 41o- bronchial epithelial cells transduced with lentivirus encoding F508del CFTR (Fig 1A and 1B) have been adopted to monitor pharmacologic correction. Despite viral expression technology and robust levels of F508del mRNA compared to WT (Fig 1A), the mutant protein in CFBE is barely detectable (Fig 1B). It may therefore be unrealistic to expect that a single corrector molecule will yield sufficient quantities of repaired F508del to approximate WT levels (Fig 1B). This is especially the case for compounds that overcome only one of the many F508del-dependent folding or quality control abnormalities.

The lack of correctable F508del CFTR protein is even more evident in primary human airway cells. Although the mRNA levels in F508del and WT epithelia are similar, the mutant protein is still barely discernible in patient samples, even when the proteins are overloaded to enhance detection of ER localized Band B (Fig 1C and 1D). Regardless of whether this deficit is attributable to increased CFTR degradation or aberrant synthesis, the paucity of F508del CFTR in primary airway epithelial cells presents a challenge to the identification of compounds that confer pharmacologic correction. Moreover, drugs that fail to overcome pronounced depletion of F508del CFTR are unlikely to achieve meaningful levels of functional rescue in patients. With this in mind, we developed compound screens that would allow us to overcome these obstacles.

### Screening for agents that increase the F508del CFTR pool

**Fig 2:**
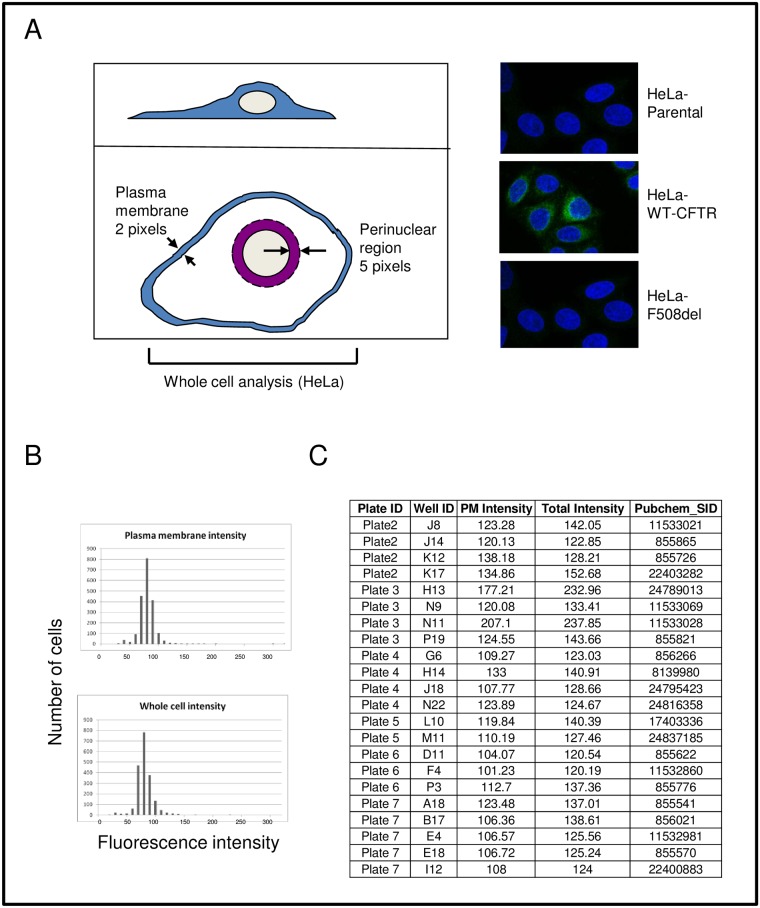
High content microscopy identifies compounds that increase the F508del CFTR pool. (A) Cartoon depicts subcellular regions of interest (ROI) for image analysis in a high throughput screen of small molecules. With this method, compounds can be identified that influence either plasma membrane or total cellular expression of F508del CFTR, with representative immunofluorescence image also shown. (B) Fluorescence intensity distribution within key regions of interest in vehicle treated samples within a high content screen of 2,000 compounds from the NIH Roadmap Molecular Libraries Small Molecule Repository. (C) PubChem identifiers for a subset of small molecules that augment total cellular and/or surface expression of F508del CFTR. Each value shown is >3 standard deviations above mean fluorescence intensity for all compounds tested.

**Fig 3:**
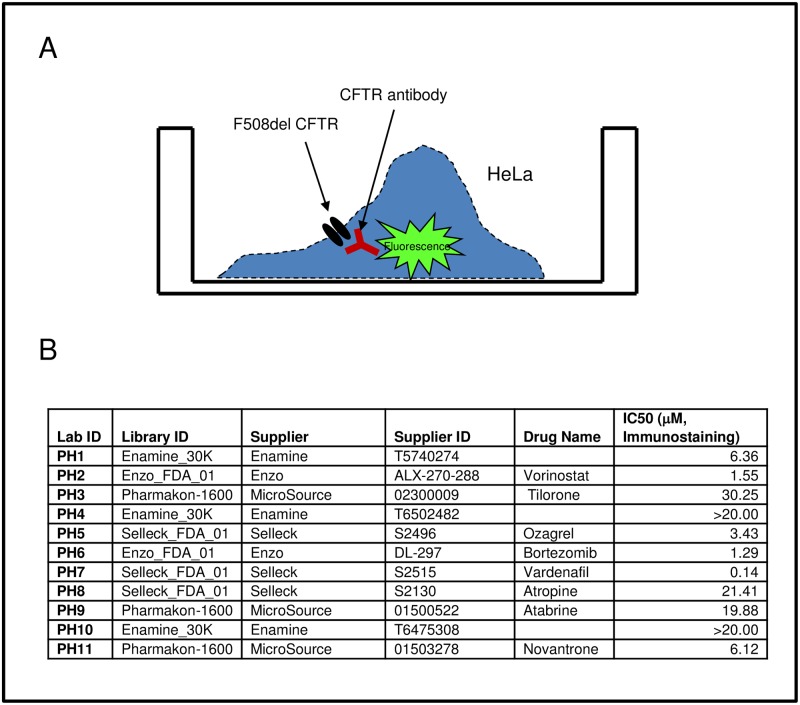
Fluorescence based total cellular CFTR measurement. (A) Cartoon depicts fluorescence based analysis in the microtiter plate assay. (B) Examples of positive hits from a screen of ~10,000 compounds using libraries including Pharmakon-1600, Enamine_30K, Enzo_FDA_01, and Selleck_FDA_01. Each value shown is >3 standard deviations above mean for all compounds tested.

**Fig 4:**
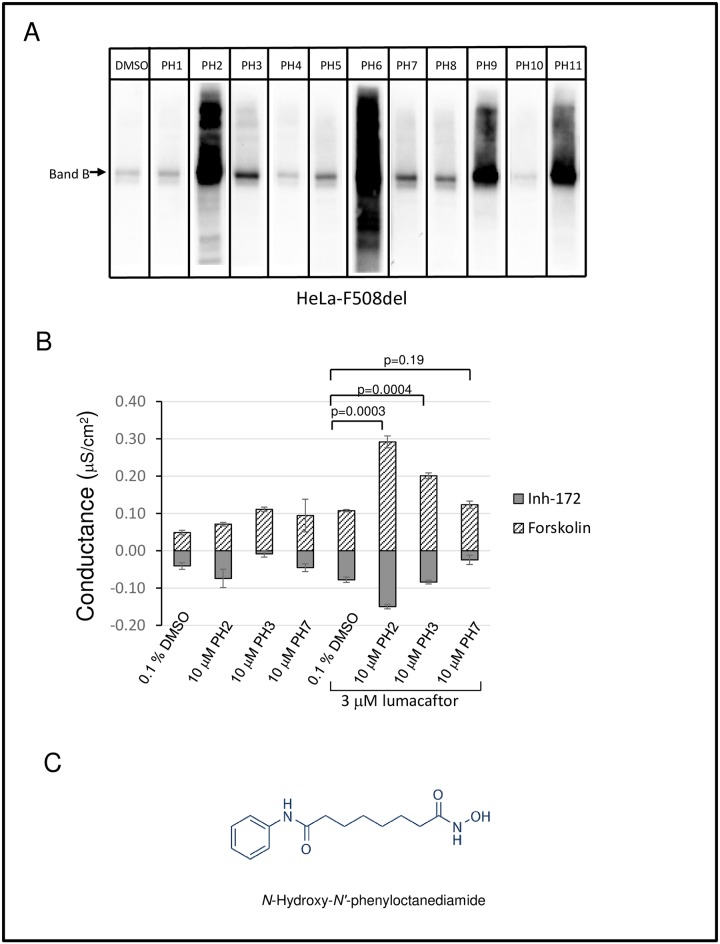
Verification of the hits using western analysis and transepithelial conductance measurements. (A) Western blots of cell lysates from HeLa expressing F508del CFTR treated for 24 hours with 10 μM primary hit compounds identified to increase total CFTR fluorescence. (B) Transepithelial conductance measurements after 24 hours incubation with selected compounds alone (10 μM), or combined with lumacaftor (3 μM). (C) Chemical structure of vorinostat.

**Fig 5:**
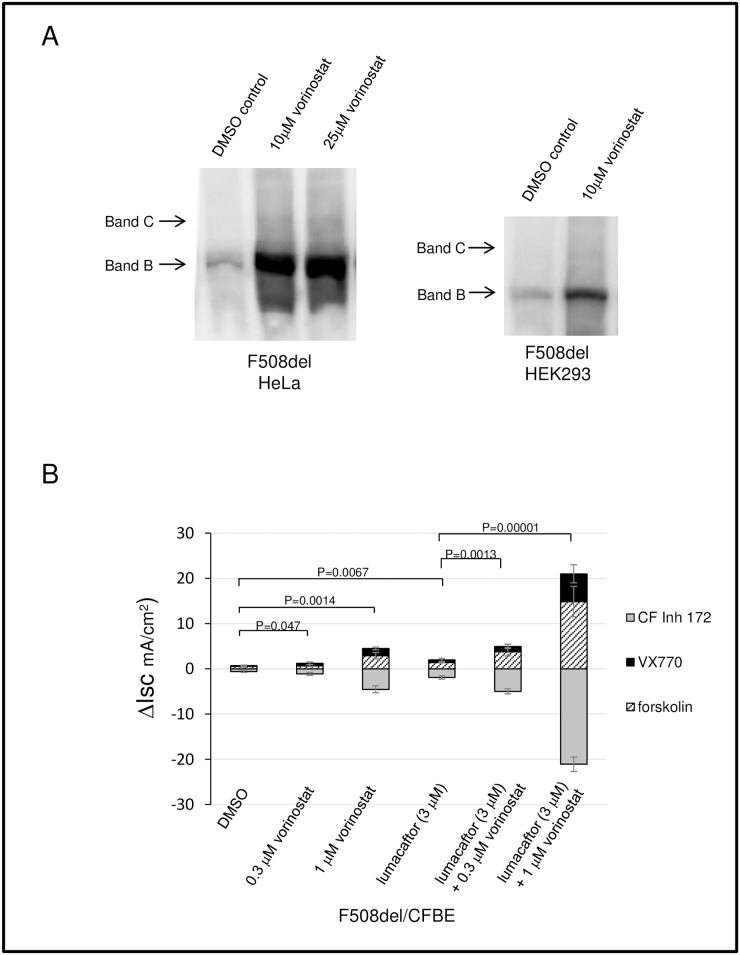
Vorinostat increases CFTR band B and augments ion channel activity in combination with lumacaftor. (A) Western blot of cell lysates treated with vorinostat from HeLa (left panel) or HEK 293 (right panel) cells. (B) Short circuit current measurements in CFBE monolayers treated with vorinostat, lumacaftor or a combination of the two compounds. After establishment of a Cl^-^ gradient and addition of amiloride (100 μM), monolayers were treated with forskolin (20 μM) and ivacaftor (VX-770, 10 μM), followed by administration of CFTR Inhibitor 172 (10 μM). N = 4 filters per condition. Error bars represent SEM with p values indicated.

Proteasome inhibition increases the levels of F508del Band B, but fails to augment rescue of the mutant protein to the cell surface [6]. We hypothesized that this phenomenon arises because F508del CFTR has become polyubiuquitinated and is aggregation-prone, thus reaching a “point of no return” beyond which refolding to the WT configuration is impossible. We developed two robotic compound library assays suitable for identifying compounds that increase early folding intermediates, i.e., the ER Band B form of mutant CFTR. In turn, early species of this type should be suitable for improved correction by compounds such as lumacaftor. In the first approach, high content confocal immunofluorescence microscopy was utilized to quantitate both plasma membrane and total CFTR. The system allows rapid data collection, and enables library screening with high resolution. This strategy has been used previously for parallel drug screening against multiple resistant HIV mutants [28]. In order to test feasibility, we analyzed ~2,000 known bioactive agents from the NIH Roadmap Molecular Libraries Small Molecule Repository (Fig 2). This library was chosen based on the range of physicochemical properties, the absence of reactive compounds, and the choice of several analogs within the library to allow for SAR development. As an indicator of the assay robustness to identify treatments that increase the F508del protein pool, the Z factor score of 0.56 +/- 0.089 (mean+/-SEM) was obtained comparing vehicle treated HeLa cells stably expressing F508del CFTR to HeLa cells expressing WT CFTR. A second, complementary strategy is shown in Fig 3, and was designed to monitor total expression of CFTR using multi-well fluorescence. This second screen further enhanced our ability to identify active compounds. For the pilot analysis, ten thousand compounds from a diverse set of libraries (Pharmakon-1600, Enamine_30K, Enzo_FDA_01, and Selleck_FDA_01) were tested at a final concentration of 25μM (Enzo, Selleck & MS Pharmakon) or 10 μg/ml (Enamine 30K diversity set). This dataset library represents a chemogenomics collection, which in principle allows us to evaluate all possible pathways that operate during CFTR biogenesis. The efficacy of the screen was determined by an obtained Z factor value of 0.63+/-0.04 (mean+/-SEM) using a proteasome inhibitor ALLN as a positive control. Under no circumstances was there a measurable effect on cell viability. Both assays identified numerous compounds that increase either plasma membrane localized or total F508del CFTR. As a secondary screen, a total of 11 compounds were tested for ability to increase F508del CFTR Band B in HeLa cells (Fig 4A). PH2, 6, 9, and 11 strongly increased steady state levels of Band B CFTR, while treatment with PH3 and 7 exhibited moderate response at 10 μM. These drugs were further tested to determine whether they augmented conductance when combined with lumacaftor in FRT cells expressing F508del CFTR (Fig 4B). PH2 (vorinostat; Fig 4C) exhibited the most significant enhancement of CFTR activity together with lumacaftor. Vorinostat was further shown to augment levels of Band B with synergistically improved activity of the CFTR corrector lumacaftor in human epithelial cells (Fig 5). The compound by itself also had a small, but statistically significant effect on F508del CFTR surface activity as measured by changes in short circuit current. These data provide additional evidence that vorinostat, which is approved for cutaneous T cell lymphoma and functions as an inhibitor of histone deacetylase (HDAC), can increase the F508del protein pool available for subsequent rescue to the plasma membrane [29].

### Repair of the F508del CFTR Pool

**Fig 6:**
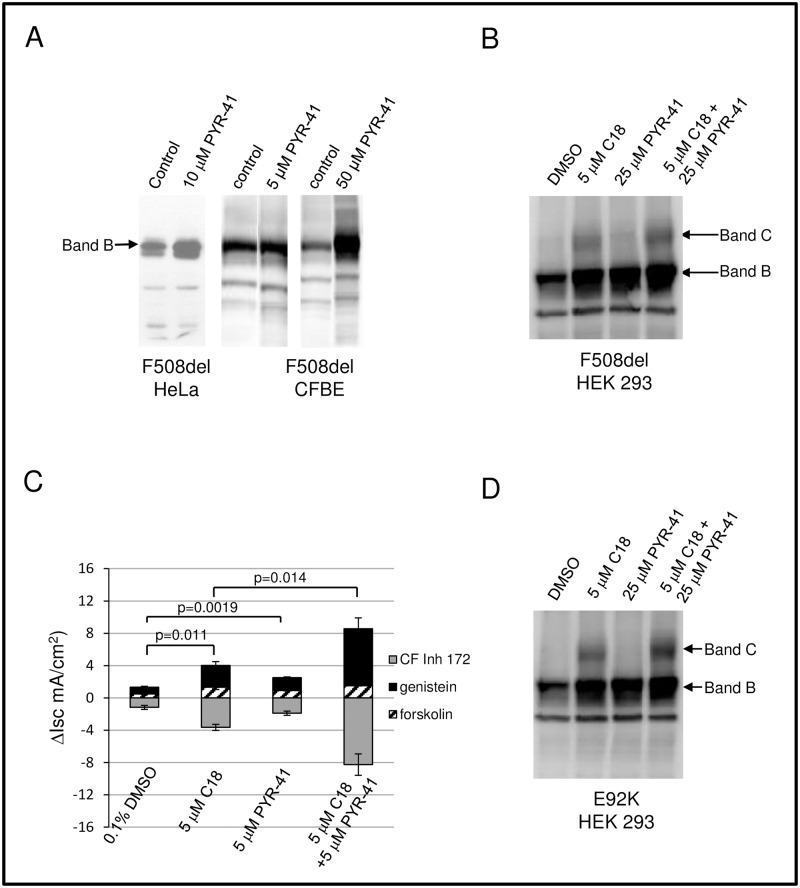
The F508del CFTR Band B pool available for correction can be increased by blocking of ubiquitination. (A, B) Western blot of HeLa, CFBE, or HEK 293 lysates treated with PYR-41 (Selleck Chemicals). (C) Short circuit current (Isc) from CFBE monolayers treated with PYR-41, C18, or a combination of both compounds monitored by modified Ussing chamber analysis. N = 4 filters per condition. Error bars represent SEM with p values indicated. The change in Isc for each perturbation is shown. Values were normalized against combined Isc changes in the DMSO control, taken as 100%. (D) Effects of PYR-41 plus C18 in HEK 293 expressing the CFTR E92K mutation.

As additional evidence that augmenting F508del Band B levels would provide a greater protein pool that might be suitable for correction, we tested whether interrupting an early, specific step in ERAD can increase the efficacy of a chemical corrector. In both cell culture and in vivo, nearly the entire pool of F508del CFTR is selected, ubiquitinated, and destroyed by the ERAD pathway [5, 30, 31]. To this end, we used PYR-41, an inhibitor of the E1 ubiquitin activating enzyme that blocks diverse functions linked to the ubiquitination pathway [32]. As anticipated, PYR-41 significantly increased the levels of Band B in three cell lines without restoring Band C (Fig 6A and 6B). While the effect of PYR-41 alone is minimal on CFTR activity as assessed in short-circuit current studies, in combination with C18, a lumacaftor structural analog that works similarly to lumacaftor in cell lines [33–35], surface correction and activity were significantly improved (Fig 6B and 6C). Importantly, the same combination therapy may be applicable to less common disease-causing CFTR alleles, such as E92K (Fig 6D, www.CFTR2.org). Individuals with this allele show classical signs of CF [36]. As above, there were no toxic effects on cell viability noted under any of the conditions employed in these experiments. The findings collectively establish that chemical inhibition of ERAD prior to substrate ubiquitination magnifies the effect of an F508del corrector.

## Discussion

**Fig 7:**
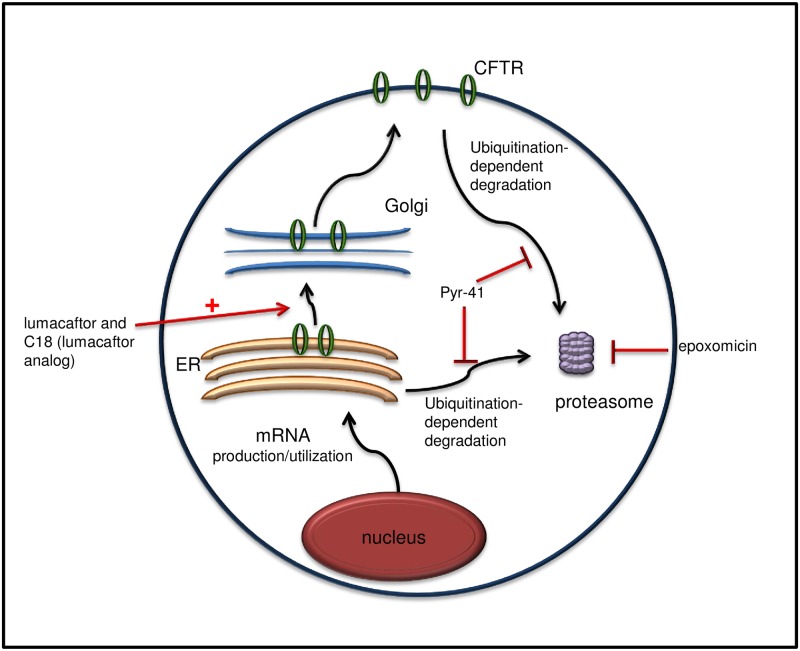
Cellular processes that influence the F508del CFTR pool.

Numerous cellular processes contribute to insufficient levels of ER localized F508del CFTR (Fig 7), including defects in mRNA utilization, enhanced ERAD, intrinsic CFTR “off pathway” folding defects, and enhanced endocytosis and lysosomal degradation [2, 15, 31, 37, 38]. At least two separate abnormalities are inherent properties associated with F508del CFTR misfolding: 1) a failed contact between the CFTR first nucleotide binding domain (NBD1) and cytosolic extensions of transmembrane α-helices, and 2) instability of NBD1, itself [14, 39]. Therefore, small molecule correction of cystic fibrosis lung disease must potentially surmount more than one of these hurdles, such as favoring NBD1 contacts (i.e., lumacaftor) [34], increasing the synthetic pool (i.e., vorinostat), or inhibiting ubiquitination (i.e., PYR-41), which will mute ERAD and potentially also lysosomal targeting and degradation.

Insufficient levels of F508del CFTR limit the effectiveness of pharmacologic repair, and may contribute to the “ceiling” sometimes ascribed to CFTR correction. In this context, we provide methods for augmenting the pool of correctable F508del CFTR Band B. The findings presented here indicate that small molecules can be identified through compound library screening to overcome the obstacle of insufficient levels of F508del CFTR at an early stage of protein folding. In turn, the increased pool of Band B then becomes available for correction by preclinical and clinical F508del CFTR correctors, such as C18 or lumacaftor, respectively [8, 17]. The assays described by the present report should also be complementary to drug screening programs utilizing CFTR surface function as an endpoint, and provide the advantage of identifying drugs that synergize with corrector molecules of numerous subtypes [34].

**Fig 8:**
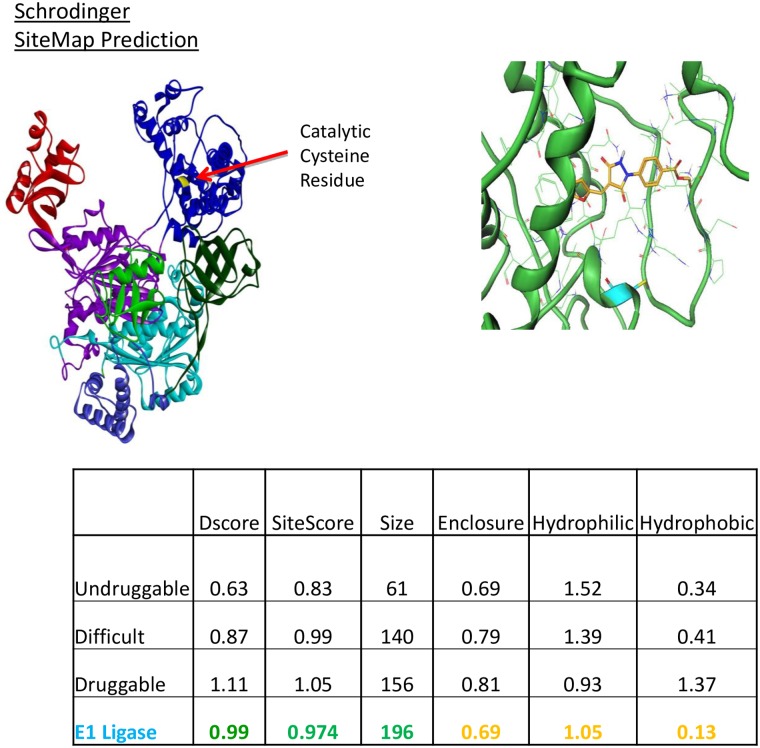
Computational chemistry as a means to identify improved E1 ubiquitin activating enzyme inhibitors. The PYR-41 binding site was modeled and suitability as a “druggable” target assessed by SiteMap [26]. Features of the predicted binding site (insert) describe a molecular target appropriate for drug optimization. The right panel shows docking of PYR-41 to the E-1 activating enzyme.

It is important to note that not all targets are druggable. Moreover, targets that have reasonable potency for small molecules often fail to translate into a clinical candidate. One estimate claims that ~60% of the small molecule drug discovery projects fail because the target is not druggable [40]. One novel aspect of our study is the ability of an E1 inhibitor, PYR-41, to exhibit synergism for F508del CFTR correction when combined with C18. In order to ascertain whether the E1 ubiquitin activating enzyme represents a druggable target, we performed a computational druggability estimate using two different approaches: a maximal affinity prediction model [25], and a well validated commercially available assessment model called SiteMap [26]. Based on these analyses, we found that E1 ubiquitin activating enzyme is druggable, according to either assessment. The maximal binding affinity model predicted a binding pocket surface area of approximately 253 Å^2^ and that an optimized ligand could bind with a K_I_ between 60–100 nM, which represents a favorable result. SiteMap analysis highlights an enzyme binding pocket that exhibits a combination of scoring features suitable for a drug-like molecule to bind (Fig 8).

Germane to this goal, blocking degradation at the proteasome with a small molecule inhibitor is not suitable for subsequent F508del CFTR correction [41–43]. In agreement with these results, we found that treatment with a more specific proteasome inhibitor (epoxomycin) drastically elevated F508del Band B, but had no beneficial effect on C18 dependent rescue [42]. These findings agree with earlier reports that F508del biogenesis is not improved by interruption of terminal events during ERAD [4, 43]. In contrast, the present experiments establish that blocking F508del degradation at a very early stage, in this case by impairing the E1 activating enzyme, confers an increase in F508del Band B well suited for folding correction (Fig 6). These data are also in accordance with findings that the siRNA based silencing of specific E3 ubiquitin ligases promotes chemical correction of F508del CFTR [44].

Pharmacologic rescue of F508del CFTR has focused on combinations of drugs that overcome distinct folding abnormalities as a means to achieve synergy. The present experiments describe high throughput protocols intended to augment the repairable CFTR pool, and reveal agents that may work by novel mechanisms. Moreover, many of the same compounds that augment the F508del Band B pool can now be tested against less common CFTR processing mutations (Fig 6D), for which corrector development has become an increasing priority [45]. The use of HDAC inhibitors such as vorinostat has previously been suggested as a means to enhance F508del correction, and identification of such agents by the pilot screen described here provide support for this overall approach [29, 46]. Further studies will be necessary to determine whether compounds such as those documented in Figs 2C and 3B act in synergy with lumacaftor (or with one another) in primary airway epithelial cells. In either case, our strategy provides a means by which drugs that improve F508del CFTR rescue can be identified and investigated in the future.

In summary, genotype/phenotype correlations among individuals with mild or atypical CF, and recent clinical data using the FDA approved potentiator of CFTR gating (ivacaftor/Kalydeco^™^), indicate that modest enhancement of F508del CFTR processing by as little as 3-fold above lumacaftor alone may be sufficient to offer substantial benefit to individuals homozygous for F508del [18]. The present studies offer a means by which correction of this magnitude might be achieved, even without the addition of a potentiator, and could be expected to increase further when combined with ivacaftor. Individuals with cystic fibrosis who carry only one copy of F508del (with a non-gating defect on the second allele) comprise a significant fraction of the CF population, and pharmacotherapy has been unsuccessful in these individuals due to insufficient levels of mutant protein available for correction [47, 48]. Increasing the foldable pool of F508del CFTR will be of particular value in the setting of F508del heterozygosity, and could address the gene-dose effect. Overall, compounds discovered by our approach will enhance efficiency of F508del correction, and help elucidate novel pathways that underlie CFTR biogenesis and maturational arrest.
